# A Satellite-Based Imaging Instrumentation Concept for Hyperspectral Thermal Remote Sensing

**DOI:** 10.3390/s17071542

**Published:** 2017-07-01

**Authors:** Thomas Udelhoven, Martin Schlerf, Karl Segl, Kaniska Mallick, Christian Bossung, Rebecca Retzlaff, Gilles Rock, Peter Fischer, Andreas Müller, Tobias Storch, Andreas Eisele, Dennis Weise, Werner Hupfer, Thiemo Knigge

**Affiliations:** 1 Department of Environmental Remote Sensing & Geoinformatics, University of Trier, 54286 Trier, Germany; bossung@uni-trier.de (C.B.); retzlaff@uni-trier.de (R.R.); rock@uni-trier.de (G.R.); 2 Luxembourg Institute of Science and Technology (*LIST*), L-4362 Esch-sur-Alzette, Luxembourg; martin.schlerf@list.lu (M.S.); kaniska.mallick@list.lu (K.M.); 3 German Research Centre for Geosciences (GFZ), 14473 Potsdam, Germany; karl.segl@gfz-potsdam.de (K.S.); eisele@gfz-potsdam.de (A.E.); 4 DLR German Aerospace Center, 82234 Weßling, Germany; peter.fischer@dlr.de (P.F.); andreas.mueller@dlr.de (A.M.); tobias.storch@dlr.de (T.S.); 5 Airbus DS GmbH, 88090 Immenstaad, Germany; dennis.weise@airbus.com (D.W.); werner.hupfer@airbus.com (W.H.); thiemo.knigge@airbus.com (T.K.)

**Keywords:** hyperspectral, thermal remote sensing, food security, satellite TIR mission

## Abstract

This paper describes the concept of the hyperspectral Earth-observing thermal infrared (TIR) satellite mission HiTeSEM (High-resolution Temperature and Spectral Emissivity Mapping). The scientific goal is to measure specific key variables from the biosphere, hydrosphere, pedosphere, and geosphere related to two global problems of significant societal relevance: food security and human health. The key variables comprise land and sea surface radiation temperature and emissivity, surface moisture, thermal inertia, evapotranspiration, soil minerals and grain size components, soil organic carbon, plant physiological variables, and heat fluxes. The retrieval of this information requires a TIR imaging system with adequate spatial and spectral resolutions and with day-night following observation capability. Another challenge is the monitoring of temporally high dynamic features like energy fluxes, which require adequate revisit time. The suggested solution is a sensor pointing concept to allow high revisit times for selected target regions (1–5 days at off-nadir). At the same time, global observations in the nadir direction are guaranteed with a lower temporal repeat cycle (>1 month). To account for the demand of a high spatial resolution for complex targets, it is suggested to combine in one optic (1) a hyperspectral TIR system with ~75 bands at 7.2–12.5 µm (instrument NEDT 0.05 K–0.1 K) and a ground sampling distance (GSD) of 60 m, and (2) a panchromatic high-resolution TIR-imager with two channels (8.0–10.25 µm and 10.25–12.5 µm) and a GSD of 20 m. The identified science case requires a good correlation of the instrument orbit with Sentinel-2 (maximum delay of 1–3 days) to combine data from the visible and near infrared (VNIR), the shortwave infrared (SWIR) and TIR spectral regions and to refine parameter retrieval.

## 1. Introduction

The thermal infrared spectral domain (TIR) has become a major source of quantitative and qualitative information within the disciplines of soil sciences, hydrology, geology, biology, oceans, and atmosphere [1,2,3,4]. This includes mapping land-surface temperature (LST) [2], sea surface temperature (SST) [4], high-temperature events (volcanoes, wildfires) [5], high spectral resolution emissivity mapping of the Earth’s surface (minerals, vegetation) [6], and gas emission detection [7]. The HiTeSEM project is a national preparatory “phase 0” study that was launched to anticipate upcoming calls from the European Space Agency (ESA) for innovative spaceborne missions targeting the world’s Earth system rationales of ESA’s Living Planet Programme [8].

The outcome of this project is a conceptual instrument design for a spaceborne hyperspectral thermal imaging instrument to contribute to solutions of the two crucial and interrelated global societal problems “human health” and “food security” [9,10]. This science case was identified based on intensive literature research and an expert user workshop in the framework of that project.

Increasing population growth, especially in urban areas, triggers the demand to extend crop areas and crop yield worldwide and at the same time to systematically monitor the status of agricultural and urban environments. However, the important information related to energy fluxes [3,4] and complex surface characteristics [11,12] cannot be quantitatively retrieved solely using orbital missions targeting the visible to shortwave infrared (VNIR-SWIR) and the microwave regions. From the TIR domain, two supplementary and complementary key variables can be retrieved in this context: LST and land surface emissivity (LSE) [4]. Both constitute physically-based properties from which further secondary information can be retrieved related to the selected science case to assess key surface properties in forests, urban, and agricultural areas, such as soil composition and conditions [11], drought-induced plant stress [13], plant discrimination [14,15], and heat fluxes and evapotranspiration [16]. This information is strongly linked to the conditions of agricultural and urban environments. This has also been recently discussed at the European Commission (EC) Copernicus Agriculture and Forestry Applications User Requirements Workshop held on 30 June 2016 [17]. The identified user demands for a related spaceborne TIR mission for agricultural services include multi-spectral TIR observations, 50 m ground resolution or better, high radiometric sensitivity, co-located multispectral imaging capabilities in the VNIR, afternoon local overpass, and daily revisit times. The demand for such a TIR mission to meet the user needs in agricultural services was recently confirmed as one of the priorities for the evolution of the European Copernicus Space Component [18].

Multispectral TIR satellite systems, such as ASTER (Advanced Spaceborne Thermal Emission and Reflection Radiometer), allow the discrimination of many surface types. However, the advent of hyperspectral TIR imagers has further extended the capability to detect and map a wide range of materials and target characteristics compared to multispectral thermal sensors [6]. Today, hyperspectral TIR data of high spatial resolution can be solely acquired by airborne imaging sensors and, thus, only for areas of limited size. Examples of airborne systems include the airborne hyperspectral imager (AHI), the Spatially-Enhanced Broadband Array Spectrograph System (SEBASS) [19], the AisaOWL [20], the Hyperspectral Thermal Emission Spectrometer (HyTES) [21], and the Hyper-Cam [22]. In this science case, spectrally highly-resolved LSE can provide very detailed information about soil surface and urban characteristics. Furthermore, the high informational content of hyperspectral data facilitates the use of machine learning algorithms for knowledge retrieval which, in turn, triggers the detection of new information and products. While the potential of hyperspectral remote sensing has been demonstrated in several studies (e.g., [11,12,13,14,15]) it has not yet been explored if a related orbital mission is feasible. One important intermediate step towards this goal will be the ECOsystem Spaceborne Thermal Radiometer Experiment on Space Station (ECOSTRESS, [23]) on the International Space Station (ISS), which aims at measuring the temperature of plants to retrieve plant physiological parameters. This mission will be mainly constrained by the orbital characteristics of the ISS.

The HiTeSEM study aims at extending airborne hyperspectral imaging towards a free flying satellite platform for global monitoring of agricultural areas, forests, and urban environments in the TIR. Towards this end the aim was to:

- Identify gaps in existing spaceborne missions to retrieve key variables that are closely related to global food security and human health;
- Assess whether related variables can be retrieved from a hyperspectral thermal instrument with the required accuracy and suggest instrument design parameters and related trade-offs; and
- To define orbit requirements and to develop a first instrument design concept.

## 2. Scientific Objective Rationale

The growing population and related consumption is still expected to increase the global demand for food and water significantly in the forthcoming decades. At the same time, climate change and the food versus fuel debate give rise to additional competition in the agricultural regions [10,24]. This, in turn, affects our capacity to produce food and, at the same time, to minimise environmental impacts and threats to human health [10,25]. In 2014, 54% of the global population lived in urban areas, approximately half of them in mega-cities with 500,000 or more people [26]. The United Nations [26] forecasts a growth of urban population to 6.4 billion in 2050. Thus, an urgent requirement arises to adequately monitor sensitive agricultural areas, the urban environment, and their surroundings.

Food security of an increasing population and sustainable agriculture is the second among the 17 global sustainable development goals (SDG) formulated by the UN [27]. Ensuring healthy lives and promoting people’s well-being is formulated as the third SDG. In addition, both aspects are integrated parts of the ESA’s Living Planet Programme.

Remote sensing has great potential to contribute to a monitoring program to support human welfare thanks to successful global satellite missions, such as Landsat, MODIS (Moderate Resolution Imaging Spectroradiometer), or ESA’s Copernicus Earth observation programme. However, precise global thermal infrared (TIR) sensing over land and coastal zones, in terms of a system affording sufficient temporal, spatial, and spectral resolution, is a missing component in current capabilities [18]. TERRA-AQUA/MODIS provides only low resolution (>1 km) TIR data. Medium spatial resolution TIR data (~100 m) are provided by TERRA/ASTER (which is close at the end of its mission life) and Landsat-8 TIRS. The local overpass times of these instruments is during the morning, but if agricultural services are to be served, LST observations in the early afternoon are preferred [28]. This excludes a direct formation, e.g., with a future Sentinel-2 mission, however, in a constellation it could complement Sentinel-2 with TIR capabilities.

## 3. Definition of Observation Requirements

From the HiTeSEM expert user workshop, the aforementioned EC Copernicus Agriculture and Forestry Applications User Requirements Workshop, and finally by literature research, user requirements for a TIR sensor were derived that were further analysed for sensor design using a dedicated end-to-end simulation tool.

### 3.1. The HiTeSEM End-To-End Simulation Tool (HeteS)

**Figure 1:**
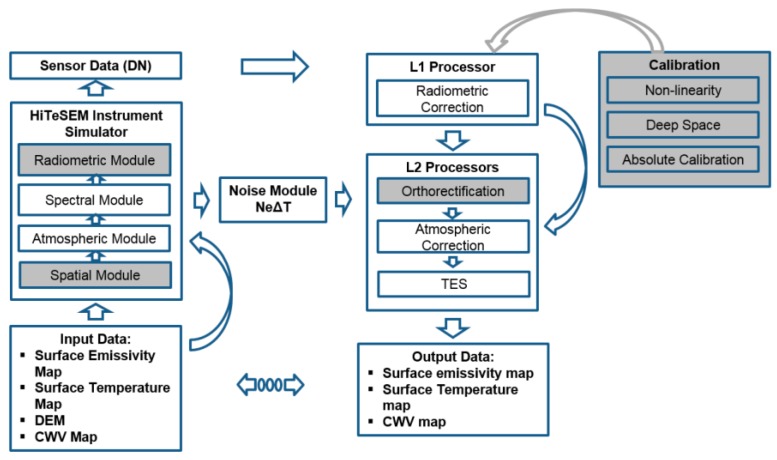
Flowchart depicting the entire HeteS end-to-end processing chain. Grey-filled modules were not applied in the study.

The definition of the sensor’s spectral sampling distance, the bandwidth, and the necessary signal-to-noise ratio (SNR) levels require a sensor end-to-end simulation tool that accurately simulates the spatial, spectral, and radiometric responses of the system, as well as the calibration and pre-processing effects. In addition, it needs to be demonstrated that the primarily key variables LST and LSE can be retrieved from a hyperspectral instrument with an accuracy comparable to existing systems, such as ASTER. The HiTeSEM end-to-end simulation tool, HeteS, is an extension from the EeteS, the EnMAP end-to-end simulation tool, and has been developed at the GFZ in Potsdam [29]. The different modules of the processing chain are depicted in Figure 1. Since the final sensor information was not available at this stage of the HiTeSEM project, the spatial, radiometric, L1, ortho, and all calibration modules (grey boxes in Figure 1) were not used within the parameter sensitivity studies.

The first part of the stripped-down version of HeteS is the generation of at-sensor radiance by the HiteSEM image simulator that requires four input data sets: (1) emissivity data, which is spectrally- and spatially-oversampled with respect to the HiteSEM final spectral and spatial sampling interval; (2) a digital elevation model (DEM); (3) a columnar water vapour (CWV) image; and (4) a surface temperature image. The atmospheric module converts surface emissivity to top-of-atmosphere (TOA) radiance data and the spectral module performs the spectral resampling, taking into account the spectral response functions (SRF) of the suggested HiTeSEM instrument. User-defined noise-equivalent-change-in-temperature (NEDT) is added in the noise module. The second part of the end-to-end simulation process comprises the pre-processing (L1/L2 processors) part. Since L1-processing is not necessary, it starts with the atmospheric transformation module that transforms the at-sensor radiance data to at-surface radiance data. The unknown columnar water vapor (CWV) is estimated in advance using a band at 7.3 µm wavelength (Section 3.1.1). The final processing step is the temperature emissivity separation module (TES). For this purpose, HeteS estimates the maximum brightness temperature currently using the two-step normalised emissivity method (NEM) algorithm [30]. In the future, in addition, an in-scene TES method shall be implemented. Related methods benefit from a high spectral resolution and narrow bandwidth channels, but that requires a good radiometric accuracy [4]. A full description of the end-to-end simulation modules and related algorithms can be found in Reference [29].

#### 3.1.1. In-Scene Water Vapour Retrieval

The radiation regime with its coupled radiance fluxes emitted from the surface and atmosphere, together with energy transfer, makes it ambitious to retrieve LST, LSE, and atmospheric parameters independently [4]. Thus, one important step in the retrieval of LST and LSE is atmospheric correction. In addition to the atmospheric temperature, the highly variable atmospheric water vapour content constitutes the most important factor in determining the atmospheric transmission and path radiance [4]. A unique feature of the HiTeSEM concept is the option for an image-based columnar water vapour (CWV) retrieval in the TIR. Simulations with HeteS have been used for band optimisation for CWV retrieval. The usage of thermal data has the significant advantage that CWV retrievals can be performed day and night, which is not feasible using water bands from the reflective part of the electromagnetic spectrum. HiTeSEM would offer the possibility to record data already from the 7.0 to 7.5 μm range. This wavelength range is dominated by the path radiance due to atmospheric absorption, which is optimal to retrieve CWV, because the surface material and its temperature do not significantly affect the recorded signal. CWV depends predominately on the surface elevation, which is normally known. Using a large database of path radiance spectra (7–8 μm wavelength range) calculated with MODTRAN5.3 that include variations of CWV (0.1–4.5 cm defined at sea level; step 0.1 cm), surface elevation (0–2500 m; step: 100 m), and sensor zenith angle (140–180°; step: 5°), retrieval models were generated using cubic splines for all possible elevation/zenith angle combinations. The investigations showed that a successful retrieval of CWV is possible in the wavelength range between 7.0 and 7.3 µm for nadir and tilt angle observations. The mean accuracy is sufficient (root-mean-square error (RMSE) < 1.0 mm for NEDT = 0.1 K) and is comparable to the accuracy retrieved by VNIR/SWIR sensors.

**Figure 2:**
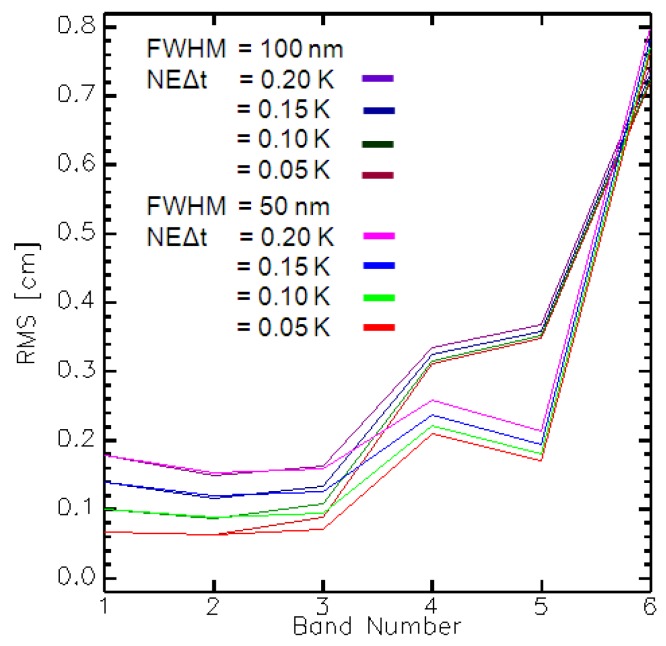
RMSE of CWV retrieval for four noise levels and seven bands (band 1 = 7.15 µm, band 2 = 7.27 µm, band 3 = 7.33 µm, band 4 = 7.46 µm, band 5 = 7.59 µm, band 6 = 7.66 µm).

Figure 2 shows the RMSE values for CWV retrieval for different noise levels and six selected spectral bands. From these results, we suggest a spectral band at 7.33 µm for CWV estimates.

#### 3.1.2. Accuracy Assessment of LST and LSE Retrievals

**Figure 3:**
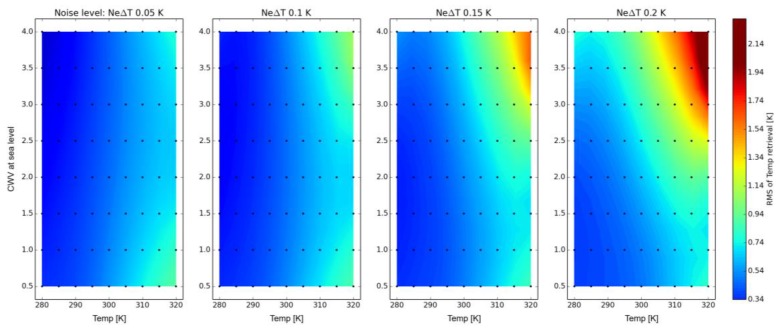
RMSE of maximum brightness temperature for 50 nm SSD/FWHM at sea-level using 0.99 emissivity as the reference.

LST is one of the key variables that is related to the TIR. To analyse the accuracy for reliable LST retrievals from hyperspectral data, a comprehensive analysis was carried out using HeteS considering five surface temperatures, eight variations of CWV, and four instrument noise levels (Figure 3).

**Figure 4:**
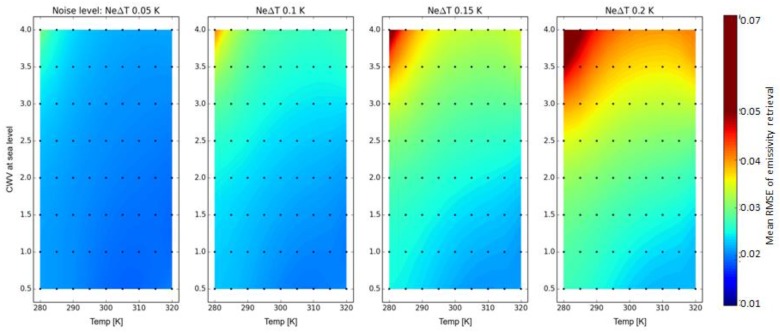
RMSE of maximum brightness temperature for 50 nm SSD (=FWHM) at sea-level using 0.99 emissivity as the reference.

The retrieved temperature values were compared with the input temperature calculating the root mean squared error (RMSE). The results are presented in Figure 4 assuming a spectral characteristic of a 50 nm spectral sampling distance (SSD) (=full width at half maximum (FWHM)). Obviously, temperature retrieval is affected especially by the accuracy of the CWV estimation and sensor noise. The RMSE also increases with higher surface temperatures, especially for NEDT values >0.1 K, indicating higher noise equivalent radiances for those temperatures. A maximum noise level of ≤0.1 K (goal: 0.05 K) is necessary to minimise all effects and to produce accurate temperature maps with an accuracy of ≤1.0 K for the investigated sensor configuration and within the envisaged temperature range.

The results for LSE retrieval simulation are depicted in Figure 4, presenting the RMSE for the combination of five temperatures, eight CWV values, and four instrument noise levels.

High RMSE values indicate that spectral spikes are present in the spectra. In contrast to the previous investigations, the low surface temperatures combined with high CWV are particularly affected. The error also increases significantly with higher noise levels. Thus, the best results are achieved for high temperatures and low CWV contents. An RMSE below 1.8% was not feasible in the simulation even for low noise levels, which can be partly explained by a non-perfect spectral alignment of input and output emissivity. Again, to achieve this accuracy a maximum noise level of NEDT ≤ 0.1 K is required.

Reliable LST and LSE estimates are the basis for the retrieval of secondary variables related to the science case that currently cannot be satisfactorily retrieved at the required temporal and/or spatial resolution in complex agricultural and urban areas using existing spaceborne capacities. The following fields of applications were analysed in more detail:

### 3.2. Mapping Minerals and Soil Surface Properties

Global monitoring of soil properties is one important demand relating to societal issues, such as food security [11]. Remote sensing in the solar reflective VNIR-SWIR domain has already demonstrated its potential for qualitative and quantitative assessment of pedogenic surface properties [11]. Among the most important parameters for evaluating and characterising soil fertility are the grain size distribution of soils, their mineral composition, and their soil organic carbon (SOC) content [31,32]. Observation of changes in these soil properties and, additionally, in water supply, allows for the assessment of the status of soil degradation—a process that may lead to nutrient loss and severe economic costs due to crop failure [33]. Soil degradation also affects ecological functions and hampers food production, especially in vulnerable regions.

SOC, clay minerals (e.g., kaolinite), iron-oxides, and -oxyhydroxides (e.g., goethite) are important for nutrient fixation and supply for crops. However, many minerals, such as quartz and feldspars, are featureless in the visible, near-infrared, and shortwave-infrared regions. Thus, for instance, mapping texture from sandy soils is limited using the VNIR-SWIR regions since the major constituent is the mineral quartz, which, on the other hand, has unique fingerprints in the TIR [11]. Thus, spectrally highly-resolved LSE adds complementary information to the optical region. Eisele and co-workers [11,31] demonstrated that hyperspectral TIR data have a greater potential for the quantitative retrieval of soil properties than the VNIR-SWIR region.

**Figure 5:**
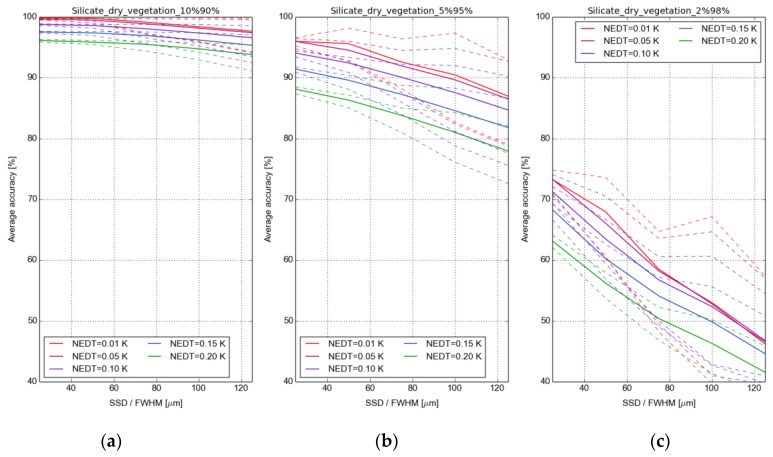
Accuracy assessment for the classification of silicates considering three mixture ratios and five noise levels: 10% mineral vs. 90% dry vegetation (**a**); 5% mineral vs. 95% dry vegetation (**b**); and 2% minerals vs. 98% dry vegetation (**c**). In the simulations it was assumed that the SSD equals the FWHM.

Mineral and soil surface characterisation benefits from a high spectral resolution, in particular for mixed material. The mixture of soils and dry and green vegetation is a common problem in remote sensing of soils. The influence of SSD on material characterisation was analysed in more detail using the HeteS end-to-end simulation tool that has been used to simulate a series of HiTeSEM-at sensor radiances from silicate and carbonate mineral spectra from the ASTER spectral library [34]. The mineral spectra were mixed at different degrees with vegetation spectra, and it was assessed whether these mineral classes can be successfully discriminated after backward simulation in the simulator. The goal was to assess the influence of the spectral resolution, noise component, and CWV content on the classification accuracy for the mixed components. As an example, Figure 5 shows the outcome of such an experiment where 15 silicate spectra were mixed with dry vegetation (three mixture ratios: 10% mineral vs. 90% vegetation; 5% mineral vs. 95% vegetation; and 2% minerals vs. 98% vegetation). In the forward simulation mode, LST was varied between 280 and 300 K and CWV was varied between 0.5 and 4 cm. The SSD was systematically varied between 30 nm and 120 nm and five noise levels were considered in the simulations (instrument NEDT = 0.01, 0.05, 0.1, 0.15, and 0.2 K). The spectrally-mixed data was further processed to surface emissivity and classified based on the spectral-angle-mapper (SAM) classifier. The quality measure is represented by the overall accuracy derived from the confusion matrix.

In case of the 10% silicate and 90% dry vegetation mixture, the mean accuracy (solid line) is less affected by noise and by the size of the SSD and FWHM because the spectral features are still distinctive. The additional dashed lines represent the worst and the best result that can be achieved by changing the centring of the bands. Increasing the vegetation fraction to 95%, the results become more sensitive towards the used parameters. The accuracy is degrading by increasing the SSD. Additionally, low noise levels can better benefit from an optimal centring of the bands compared to higher noise levels. Finally, a strong degradation of the accuracy becomes visible by increasing the SSD in case of a 98% vegetation mixture. These results demonstrate the potential of the LWIR spectral range for identifying mixed minerals and mapping their spatial distribution. From these, and comparable, HeteS experiments a minimum SSD of 100 nm (goal: SSD = 80 nm) for the assessment of complex surface properties is suggested for HiTeSEM and an instrument NEDT@300K of 0.05–0.1 K. Considering other system characteristics, the demand for a high revisit time can be relaxed to a period of ca. one month compared to other applications, such as the retrieval of evapotranspiration (Section 3.3), since the dynamics of soil properties is considerably lower. Advantageous for monitoring soils in heterogeneous agricultural areas is a GSD ~50 m [18]. Day/night revisit capability <3 days is a further desired HiTeSEM feature, as thermal inertia is related to surface soil water content [4].

### 3.3. Retrieval of Evaporation and Plant Water Stress

Food production depends on the vigour and health of a crop. Therefore, it is important to detect potential risks on agricultural sites in an early stage. Information that can be retrieved from existing spaceborne missions operating in the VNIR-SWIR and microwave range comprise the assessment of crop production, yield estimation, crop phenology, stress situations, gap fraction and leaf area index, pigments, and soil and leaf water contents [35]. However, there are two critical questions that cannot be satisfactorily answered without taking the TIR domain into account [36]: when to irrigate the crops and how much water should be applied during sensitive growth stages? In particular, in many semiarid and humid regions of the world, irrigation is of crucial importance to avoid water stress and, thus, to obtain a maximised crop yield. It is thus of critical importance for managers responsible for planning and management of water resources to understand the spatial and temporal variations in crop temperature [37]. Particularly for complex agricultural areas, this requires a sensor concept affording a good GSD (<60 m), a short revisit time (<3 days), and day/night follow-up observation capability.

A number of thermal soil-vegetation-water indices have been postulated over the last 30 years in order to detect crop water stress conditions that are reviewed by Moran et al. [28]. However, for characterising the crop water requirement it is necessary to quantify the total amount of water lost through actual crop evapotranspiration (ET) [4]. In general, ET can be determined through remote sensing by the direct or indirect approaches [4,38]. In the first case, actual crop ET is quantified through modelling the surface energy balance (SEB) fluxes, thereby incorporating LST. In the indirect case, crop ET is quantified by scaling the potential ET with an empirical crop factor, thereby incorporating VNIR images. The direct approach allows relatively accurate ET retrieval [39,40] and thus this is the preferred approach for HiTeSEM.

One direct technique is the surface temperature initiated closure (STIC) model that aims to physically integrate the radiometric surface temperature into the Penman-Monteith equation to estimate the terrestrial surface energy balance fluxes, and that does not require empirical parameterisation of the aerodynamic and canopy conductances [16]. The conductances are analytically estimated by constraining them through LST, radiation, and meteorological variables.

**Figure 6:**
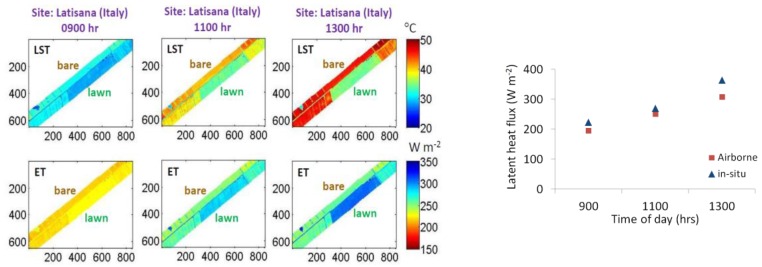
Retrieval of LST and ET from vegetated and non-vegetated areas in Latisana (left) and comparison of the airborne latent heat flux with in situ eddy covariance data. Axes depict pixel numbers (pixel size = 0.5 m) with the origin in the upper right image. The shown image strip is about 80 m wide and 600 m long.

One key question in the HiTeSEM study was to assess if ET can be reliably derived in situ from hyperspectral TIR data. For estimating the ET rate, LST is needed with an absolute uncertainty of ≤1 K for agricultural applications [41]. Figure 6 shows the outcome of an airborne campaign in Latisana (Northern Italy) (2016) using a Telops Hypercam imaging instrument. The instrument NEDT of the Hypercam is 0.05 K @ 300 K, but the signal is degraded through atmospheric and other effects if the instrument is used on an airborne platform. The hyperspectral TIR images were acquired with a spectral sampling distance of 60 nm at a survey height of 1430 m above ground. A “blackbody fit” approach [42] was performed to retrieve LST from spectral radiances and the STIC model (STIC1.2) has been used to retrieve latent heat fluxes from lawn plots and from bare soil. Results were compared with ground-based eddy covariance data.

The evaluation results demonstrate that ET can be successfully retrieved using an operational airborne hyperspectral thermal imaging instrument. It is expected that it can also be achieved using a satellite platform, provided that the instrument characteristics, in particular the noise contribution, are comparable. LST and ET contrasts are greater in the early afternoon in the vegetated plots compared to the morning period. Thus, an ideal local overpass time of a thermal satellite mission is 1300–1400 hr. The comparison confirms a good correspondence between STIC-retrieved ET data and in situ measurement, although a systematic underestimation of real ET values can be observed. This demonstrates that reliable ET estimates are feasible using hyperspectral TIR data. The related accuracy requirements for absolute LST retrievals using STIC are within 1 K.

Reliable direct and indirect ET retrieval benefits from the assessment of biome types, species composition, and relevant canopy biophysical variables, that can be derived, e.g., from Sentinel-2 or Landsat-8 data. Thus, a good correlation of the HiTeSEM orbit with Sentinel-2 is mandatory (1–3 days). In addition, the quantitative retrieval of ET and the derivation of drought sensitivity indicators [13] from TIR data could establish an important link between HiTeSEM and the forthcoming ESA FLEX Earth Explorer mission, which addresses the photosynthetic activity of vegetation at the global scale.

In a recent study [13], spectral emissivity measured at short distance over plant canopies was shown to be more sensitive than spectral reflectance to water deficit stress. However, the required accuracy for emissivity of 0.5% to discriminate drought stressed and healthy canopy will be not be achieved according to the HeteS simulations (compare Section 3.1.1). The main challenges are related to the signal-to-noise, atmosphere correction, and TES, and, most importantly, scale effects related to increasing observation distance, such as mixed pixels, scattering, re-radiation, and cavity effects [14].

### 3.4. Urban Environments and Human Health

Thermal remote sensing has a high potential in the discipline of environmental epidemiology and its application in human health. The TIR spectral region can provide necessary information for the assessment of disease expansion or provide early warning systems with necessary data. The analysis of surface crop temperatures is useful for the differentiation of microhabitats as inputs into predictive climate modelling [43,44]. Especially LST and Sea Surface Temperature (SST) of coastal waters or river estuaries, and information of soil moisture with high accuracy is required to estimate the probability of an epidemic outcome for a disease [44,45].

One other relevant subject related to human health is the assessment of urbanisation and megacity development and how this affects the local, regional, and global thermal environment. Thermal remote sensing is used to assess urban heat islands (UHI), to model urban surface atmosphere exchanges, and to analyse the complex three-dimensional structure of the urban canopy layer (UCL) [12,46,47]. A characterisation and classification of the complex UCL can benefit from the combination of VNIR-SWIR and TIR, as these spectral domains are highly complementary for urban monitoring [12]. The demand for spatial resolutions are even higher than for monitoring agricultural areas due to the higher degree of urban complexity. Thus, a spatial resolution of ~20 m is suggested [48]. Similar to the characterisation of minerals and soils, mapping the UCL benefits from a spectrally high monitored LSE [12].

## 4. Sensor Requirements

Since the scientific demands result in trade-off effects, the system requirements ask for a compromise in several aspects. The demands of small a GSD and high spectral sampling distance can be fulfilled using two instruments:

1. A spectrometer with a 60-m GSD and a broadband TIR-imager with a 20-m GSD.
2. Global coverage in the nadir direction would result in a limited repeat cycle (~one month). Shorter revisit times in the range of 1–5 days can be achieved only by accepting off-nadir observations for a limited number of geographical areas. This requires designing the HiTeSEM instrument as a pointing device with global accessibility. One common demand for all aforementioned applications is a geometric on-board calibration to enable a geo-localisation accuracy with sub-pixel accuracy especially for homogeneous targets without usage of ground control points.

This results in the following main instrument requirements (Table 1).

There are three major demands concerning the orbit requirements (Table 2): (a) good correlation with Sentinel-2; (b) global coverage in the nadir orientation; and (c) day-night follow-up observation capability. These requirements are conflicting to some extent and the orbit cannot be optimised for each.

A suitable orbit with a 47-day repeat cycle and quasi-global accessibility in the nadir orientation was identified, which combines the needs for global accessibility in the nadir orientation and for the challenging day-night follow-up observations and the required Sentinel-2 coordination, while limiting the altitude to about 500 km. This orbit would allow at least one global assessment of targets with lower temporal dynamics, such as bare soils, mega-cities, and geology/mineralogy during a mission lifetime of at least five years. In contrast, monitoring dynamic targets, such as agricultural areas, requires flexible pointing capabilities to obtain temporal revisit times < 5 days (off-nadir) and day-night follow-up observations with a time lag of <2.5 days (10°–20° off-nadir). This can be achieved by a flexible (movable) instrument design. The off-nadir angle to achieve this demand is less than 30°. Follow-up observations (day/night and day/day) would be possible for geographic locations between 60° N and 60° S.

There is a strong requirement for HiTeSEM to be synchronous with Sentinel-2 observations to combine data from the VNIR-SWIR and TIR domains. In particular, vegetation properties and condition are best predicted with a combination of VNIR-SWIR and TIR sensors or with VNIR-SWIR data alone. To raise the impact of HiTeSEM, the difference in acquisition time between these two missions are <2.5 days in advance or after. Due to the nature of the sensor, a sun-synchronous orbit is mandatory, with 14:00 LTAN or LTDN.

## 5. HiTeSEM Instrument Concept

The HiTeSEM instrument concept has been developed by Airbus DS GmbH. It is based on the combination of spectrometer (main instrument) and high-resolution imager (secondary instrument) functions into one single optical instrument, with the major characteristic being that the spectrometer works essentially as an imager. This is mainly feasible due to the assumed availability of two technologies:

- A linear variable filter (LVF) as the dispersive element for the spectrometer; and
- Design flexibility of the mercury cadmium telluride (MCT) detector and standard readout integrated circuit (ROIC)

**Figure 7:**
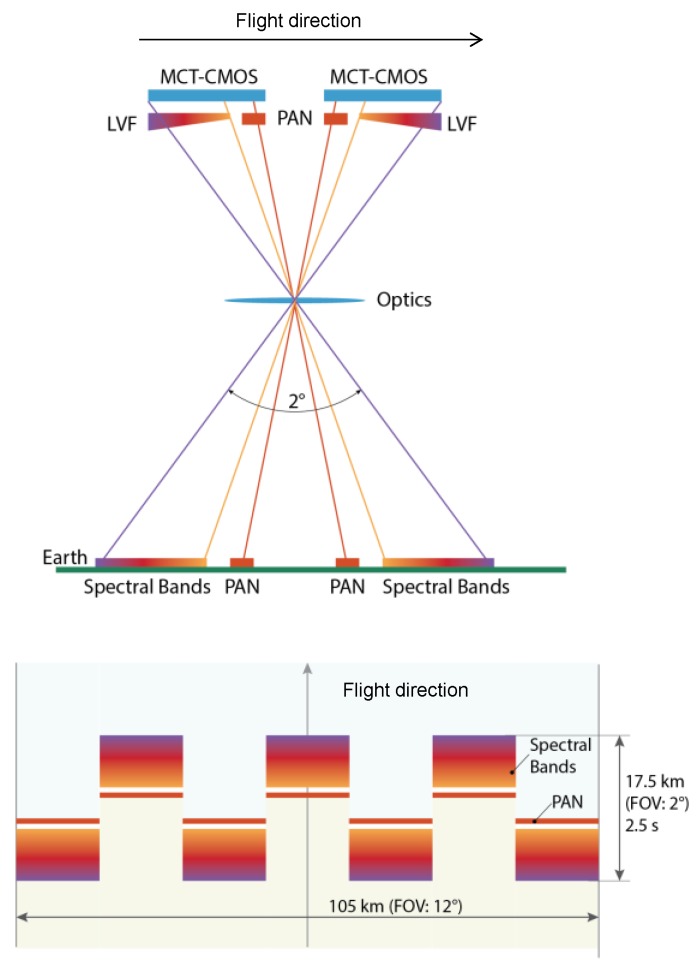
Instrument observation principle.

The spectrometer and imager functions are integrated into a single focal plane assembly (FPA) and eliminates the need for an additional conventional spectrometer module, employing gratings or prisms, and for complex FPA arrangements (e.g., folding and beam-splitting optics, separate detector/ROIC designs). The scanning concept is of the push-broom type with the following FPA geometry: spatial pixels are located in across-track direction, whereas spectral pixels are located in the along-track direction implemented by the LVF integrated onto the detector/ROIC device. With the push-broom function, the complete spectrum of one ground sample is acquired over time as the instrument moves along-track, as shown in Figure 7. The imager function is implemented by additional band-pass filter(s) adjacent to the LVF on the same detector/ROIC device. In the following, this is called the detector module.

Due to detector/ROIC development size limitations, a staggered arrangement of seven detector modules is implemented to cover the field of view (FOV) across-track.

The instrument concept is summarised in Table 3.

The ROIC architecture allows the implementation of dedicated pixel pitch sizes for the spectrometer function and the imager function on the same ROIC. The base pixel pitch is 18 µm and this is directly used for the imager function. For the spectrometer function, macro pixels are implemented based on 4 × 4 pixels resulting in a pitch of 72 µm. The relevant image acquisition parameters, such as full-well capacity, integration time, and readout frequency, are correspondingly scaled.

The optical design of the telescope is a reflective system based on a wide-field Korsch telescope concept. On-board radiometric calibration is implemented with a calibration assembly consisting of a movable mirror that folds a blackbody radiator (~330 K) into the optical path. Calibration encompasses detector flat-field measurements for pixel defect and pixel non-uniformity characterisation and absolute radiometric calibration. Pixel dark-signal measurements are performed by pointing the S/C toward deep space.

The optimum operational temperature of the MCT detectors is roughly 70 K. The complete FPA is mounted on a cold-finger and enclosed in a Dewar, which is cooled to this temperature by a cryo-cooler connected to a dedicated radiator for dissipation into cold space. A cold-stop at the entrance of the Dewar prevents thermal stray-light from the telescope and from structural components from entering the optical path.

The geometric on-board calibration is ensured by three components, (a) an Astro APS (Active Pixel Ensor) star tracker; (b) a dual-channel Global Positioning System (GPS) /Galileo receiver; and (c) the Astro 120 fibre optical gyroscope. The combination of these three high-end instruments enable a geolocation accuracy of better than 12 m (0.2 pixel) without usage of ground control points.

The main final parameters of the instrument are summarised in Table 4. It demonstrates that the major instrument requirements (Table 1) can be realized. The spectrometer and the imager share the same optical parameters. The distinction between the two instruments is only at the detector module level with different optical filter and pixel architecture (and related electrical) parameters. The derived HiTeSEM platform requirements are globally seen as the standard for a low orbit Earth observation mission; thus, the use of a standardised, in most parts recurring, platform is attractive to minimise cost and risk. The instrument dimensions, mass (of about 220 kg), and I/F requirements make it a perfect fit for the Airbus DS AS250/400 platform family.

## 6. Conclusions

A hyperspectral TIR-mission would allow for the retrieval of key variables in the framework of the two important global problems of food security and human health at high spatial, spectral, and temporal resolution. The HiTeSEM study could demonstrate that a hyperspectral thermal mission is feasible. The primary variables retrieved from hyperspectral TIR data include LST, thermal inertia, and high-resolution spectral emissivity features from the Earth’s surface and the atmosphere; they can be estimated from space with spatial and temporal resolution that are required for the estimation of many secondary variables relevant to the selected science case. The presented outcomes are preliminary in the sense that a full science traceability matrix, including how accurate secondary variables can be retrieved from spaceborne LST and LSE, science requirements, and instrument/mission requirements have not yet been developed. If at all continued, this would be the topic for a further project phase.

In combination with other missions, in particular with Sentinel-II, HiTeSEM would enable the analysis of dynamic processes in heterogeneous landscapes and to monitor human-environmental interactions. It may also contribute to the observational requirements for security mapping by its capability to observe surface phenomena both day and night at high spatial resolution.

A new multi- or hyperspectral thermal satellite mission is mandatory in the mid- or long-term, since the design life of the platforms Terra (with the ASTER instrument) and Aqua are far exceeded and beyond their shutdown period. HiTeSEM could play an important part to ensure continuing global satellite TIR observations as part of data continuity. HiTeSEM would also support other programs, such as the Copernicus observational system, by acquiring information on soil composition and dynamics, vegetation state, surface energy fluxes, and geophysical phenomena at different scales and, therefore, increase the understanding of how changes originating at regional and local levels produce effects on the global scale.

## Figures and Tables

**Table 1:** Proposed technical TIR spectrometer and imager requirements.

Parameter	Spectrometer	Broadband Imager
Spectral range	7.2–12.5 µm	7.2–12.5 µm
No. of channels	30–75	2
Wavelength accuracy	FWHM/40 –	
	FWHM/20	
NEDT (instrument)	0.05 K–0.1 K @ 300 K	<0.01 K @ 300 K
Absolute ΔT	1 K @ 300 K	1 K @ 300 K
Thermal range	250–340 K	250–340 K
Radiometric quantisation	14-bit	14-bit
Ground sampling distance at nadir	60 m	20 m
Dynamic range	Observations of targets of up to 330 K temperature without pixel saturation.	
Integrated energy	≥50%.	
Radiometric on-board calibration	Yes	
Swath width	60–120 km	60–120 km

**Table 2:** Orbit requirement and constraints.

Parameter	Performance	Constraints
Type of orbit	Sun-synchronous low Earth orbit with a local time of 14:00 h LTAN (Local Time on Ascending Node) or LTDN (Local Time on Descending Node)	
Follow-up observations	Day/night follow-up observation period: within 2.5 days	Only feasible for selected targets
		Only off-nadir view (10°–20°)
	Day/day follow-up observation period: 1–5 days	Only feasible for selected targets
		Only off-nadir view (20°–30°)
Repeat cycle	Global coverage/accessibility in nadir view: 47 days	
Nadir access	Global	
Correlation of VNIS/SWIR observations with TIR	Sentinel-2 coordination <2.5 days	
Altitude	<500 m	Aperture max. 30 cm

**Table 3:** HiTeSEM instrument concept.

**Instrument**	Scanning Thermal Infrared Imaging Spectrometer with an Integrated High-Resolution Imager
**Optic Design**	Wide-Field Korsch Telescope
**Dispersive Element**	Linear Variable Filter (LVF) (interference filter)
**Detector**	Seven staggered detector-modules consisting of hybridised MCT detectors with CMOS ROIC, LVF, band-pass filters (about 20 mm × 10 mm)
**Thermal Control**	Detector module actively cooled to 70 K, warm optics, dedicated instrument radiator, FPA dissipation <300 mW
**Structural**	All SiC (silicon carbide) homothetic design; optical elements and focal plane mounted on optical bench; isostatic mounting on S/C
**Calibration**	On-board blackbody @330 K; deep space view by S/C

**Table 4:** HiTeSEM Instrument parameters.

Parameter	Spectrometer	Broadband Imager
Spectral range	7.33 µm (for CWV retrieval)	8.0–10.25 µm
	8.0–12.5 µm (75 channels)	10.25–12.5 µm
Spectral resolution	60 nm	Pan-chromatic
Swath width	105 km (±6°)	105 km (±6°)
FWHM	1.25 × SSD	
GSD	60 m	20 m
Optical aperture	250 mm	250 mm
Focal length	650 mm	650 mm
F-number	2.6	2.6
Pixel pitch	72 µm	18 µm
Number of pixel across-track		
Without overlap (effective)	1850	7400
With overlap (per detector)	1946	7784
Integration time	7.8 ms	0.6 ms
Framerate	127 Hz	508 Hz
Detector quantum efficiency	0.8	0.8
NEDT (Instrument)	0.033–0.043 K	0.06 K
